# Supplementary material for: Elastic properties and tensile strength of 2D Ti3C2Tx MXene monolayers
Source: Nat Commun. 2024 Feb 21;15:1566. doi: 10.1038/s41467-024-45657-6 (PMC10879101; doi:10.1038/s41467-024-45657-6)
Supplement: Supplementary file 2 — Description of Additional Supplementary Files [file 41467_2024_45657_MOESM2_ESM.pdf]

DESCRIPTION OF ADDITIONAL SUPPLEMENTAR FILES DOCUMENT

Title: Supplementary Movie 1:

In situ tensile testing of the Ti3C2Tx MXene Monolayer sample until fracture (with low Pt deposition).

Title: Supplementary Movie 2:

In situ tensile testing of the Ti3C2Tx MXene Monolayer sample until fracture (with high Pt deposition).
