# Supplementary material for: Elastic properties and tensile strength of 2D Ti3C2Tx MXene monolayers
Source: Nat Commun. 2024 Feb 21;15:1566. doi: 10.1038/s41467-024-45657-6 (PMC10879101; doi:10.1038/s41467-024-45657-6)
Supplement: Supplementary file 5 — Supplementary Information [file 41467_2024_45657_MOESM5_ESM.pdf]

Supplementary Information

Elastic Properties and Tensile Strength of 2D $Ti_3C_2T_x$ MXene Monolayers

Chao Rong [a, b, c], Ting Su [a, b, c], Zhenkai Li [a, b, c], Tianshu Chu [a, b, c], Mingliang Zhu [a, b, c], Yabin Yan [a, b, c, *], Bowei Zhang [a, b, c, *] & Fu-Zhen Xuan [a, b, c, *]

[a] Shanghai Key Laboratory of Intelligent Sensing and Detection Technology, East China University of Science and Technology, Shanghai 200237, P.R. China.

[b] Key Laboratory of Pressure Systems and Safety of Ministry of Education, East China University of Science and Technology, Shanghai 200237, P.R. China.

[c] School of Mechanical and Power Engineering, East China University of Science and Technology, Shanghai 200237, P.R. China.

*Corresponding to Email: Y. Y. (yanyabin@ecust.edu.cn), B. Z. (boweiz@ecust.edu.cn) and F. X. (fzxuan@ecust.edu.cn)

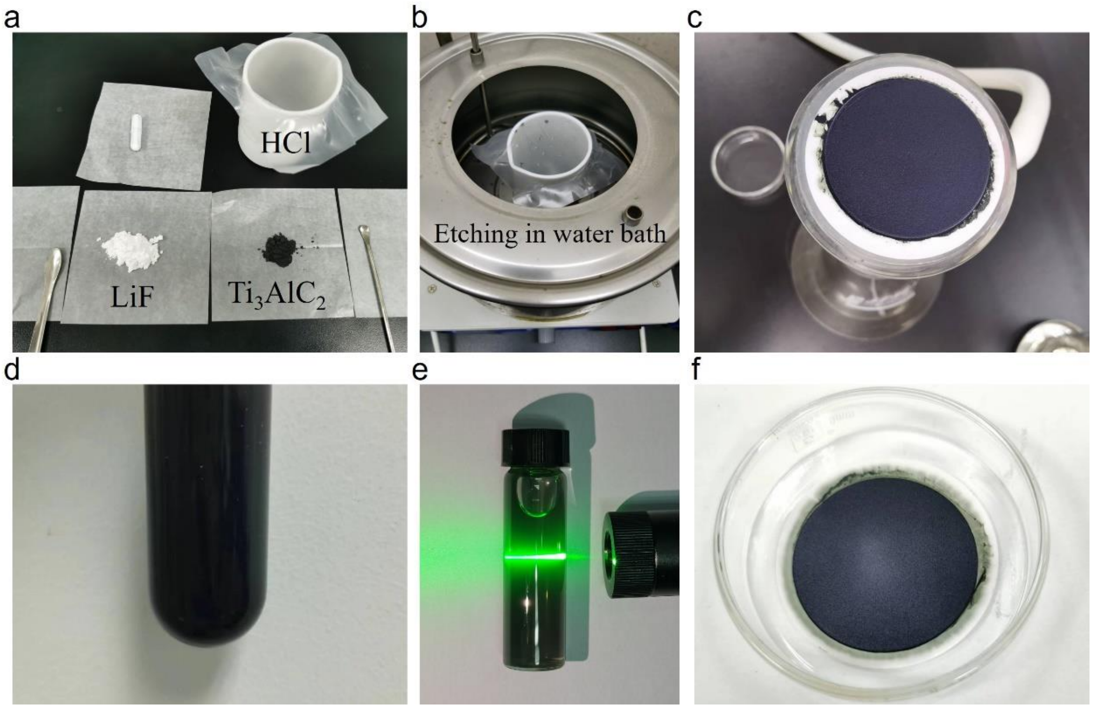

Supplementary Fig. 1 Synthesis of monolayer $Ti_3C_2T_x$ MXenes nanosheets. (a, b) Selective etching of Ti_3AlC_2 MAX phase with mixture of HCl and LiF. (c) Multilayer $Ti_3C_2T_x$ MXene film after vacuum filtration. (d) Multilayer $Ti_3C_2T_x$ MXene solution. (e) Dark green monolayer $Ti_3C_2T_x$ MXene suspension, which undergoes the Tyndall effect by laser irradiation. (f) Monolayer $Ti_3C_2T_x$ MXene film after vacuum filtration.

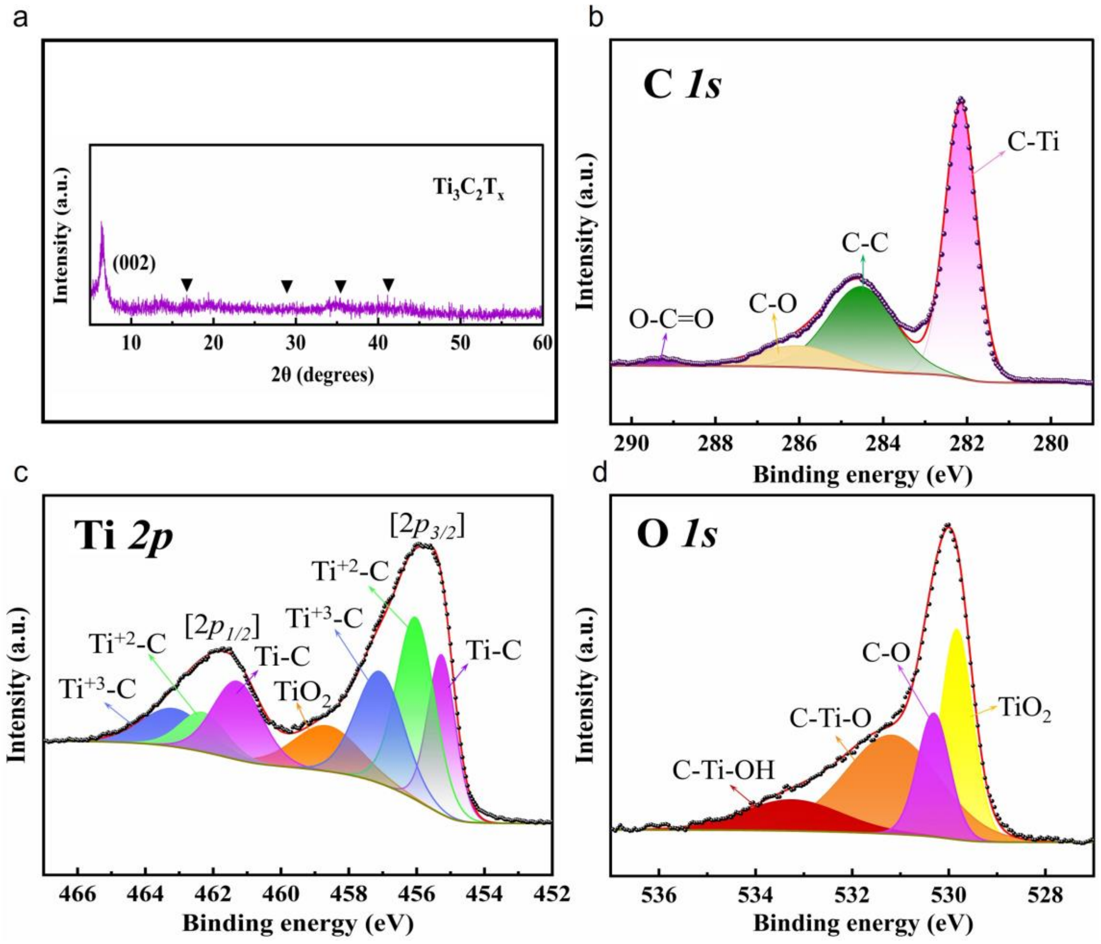

Supplementary Fig. 2 Characterization of the prepared monolayer $Ti_3C_2T_x$. (a) XRD pattern of $Ti_3C_2T_x$. High-resolution XPS spectra of the (b) C *1s*, (c) Ti *2p*, (d) O *1s* from $Ti_3C_2T_x$.

Supplementary Fig. 2b, c, d show high-resolution C *1s*, Ti *2p*, and O *1s* XPS spectra of the $Ti_3C_2T_x$. The C *1s* peak was fitted using four components located at 282.2, 284.6, 286.2, and 289.3 eV, corresponding to the C-Ti, C-C, C-O, and O-C=O bonds. The bindings of Ti $2p_{3/2}$ at 455.3, 456.0, 457.1, and 458.7 eV are attributed to the Ti-C, Ti^{+2}-C, Ti^{+3}-C bond, and Ti^{4+} ions ($TiO_{2-x}F_x$), respectively. Similarly, the main O *1s* core-level peaks at 529.8, 530.3, 531.2, and 533.3 eV are related to TiO_2, C–O, C-Ti-O, and C-Ti-OH in order[1,2].

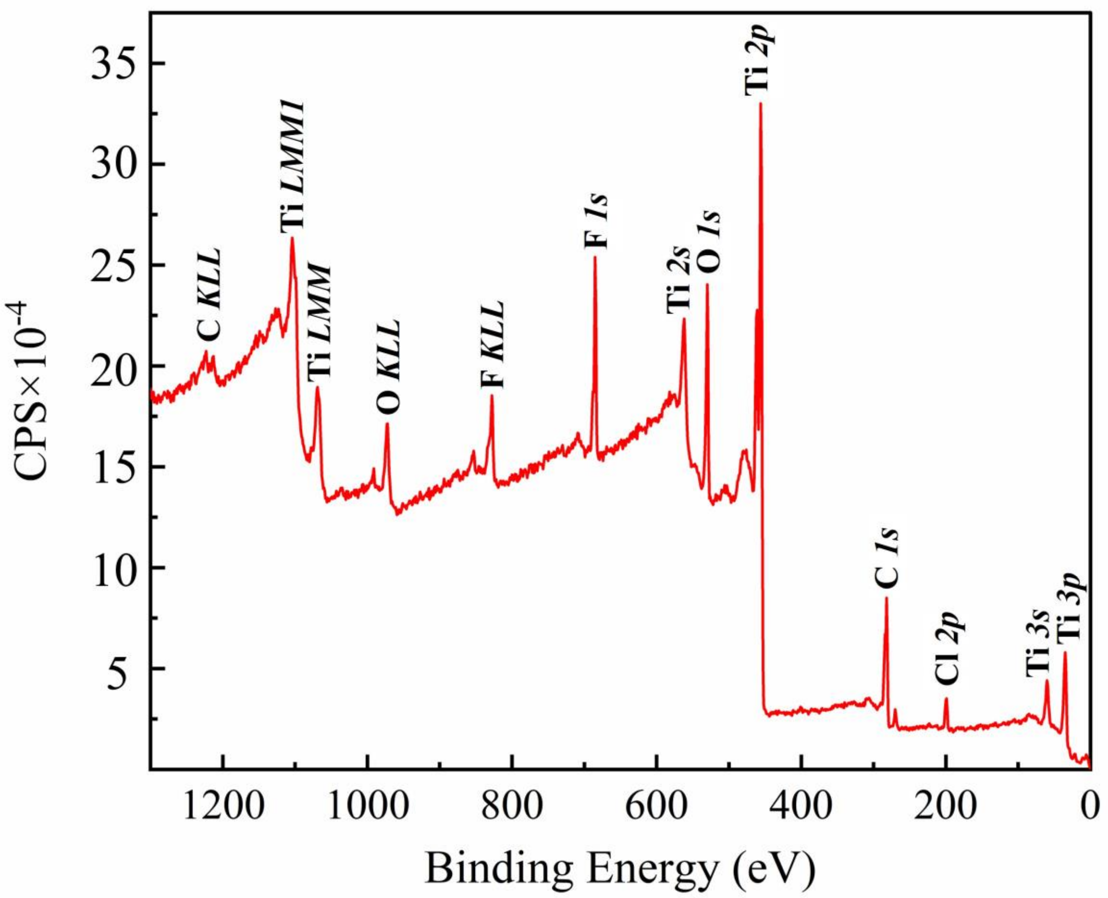

Name	Peak BE	Height CPS	FWHM eV	Area (P) CPS.eV
Ti *2p*	455.85	243933	3.54	1335697.77
O *1s*	530.05	96897.72	3.28	368686.88
F *1s*	685.02	88590.1	3.03	360617.09
C *1s*	282.37	55519.51	2.95	264481.58
Cl *2p*	199.44	15644.18	3.59	70262.91

Supplementary Fig. 3 Full XPS spectrum of the prepared monolayer $Ti_3C_2T_x$ MXene. F *KLL*, O *KLL* and C *KLL* are the Auger electron peaks of F, O and C. Ti *LMM* and Ti *LMM1* are the two Auger electron peaks of the Ti.

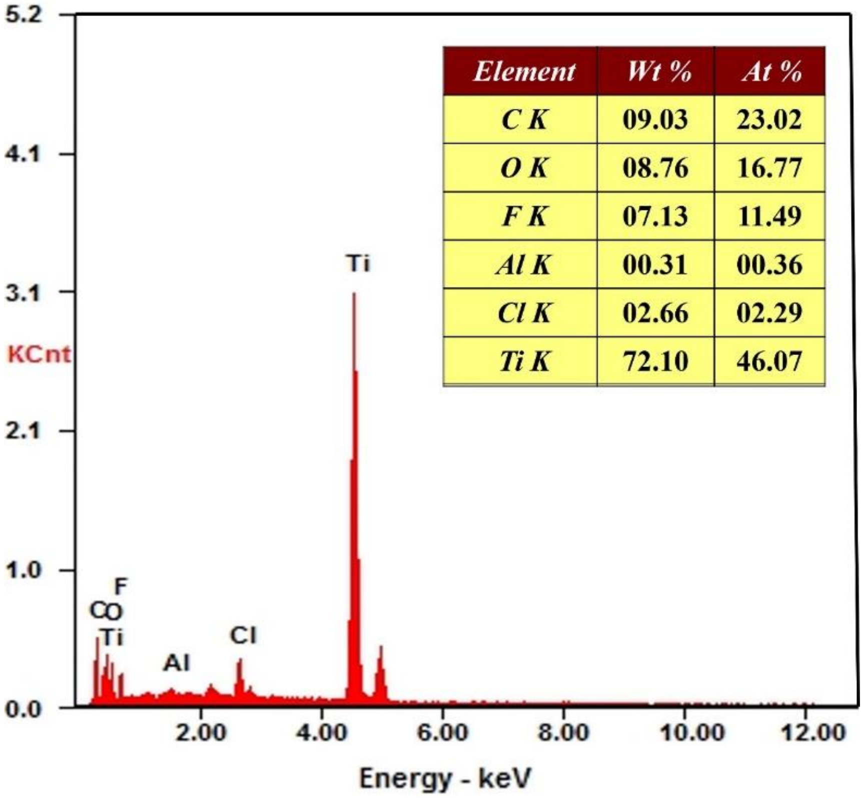

Supplementary Fig. 4 Quantitative EDX analysis of monolayer $Ti_3C_2T_x$ MXene film after vacuum filtration.

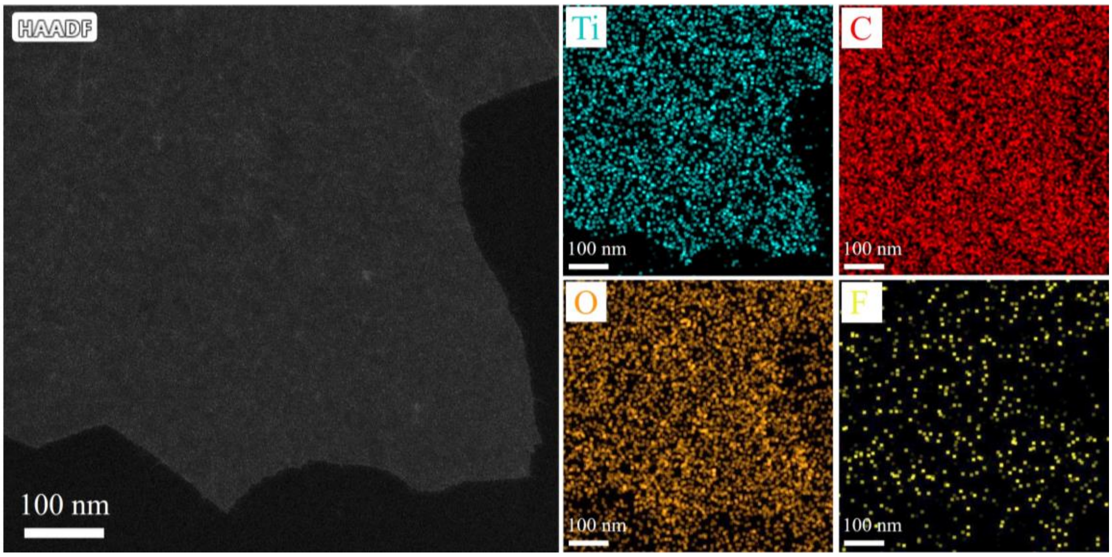

Supplementary Fig. 5 HAADF and elemental mapping images of monolayer $Ti_3C_2T_x$ MXene nanosheet.

Transmission electron microscopy (TEM) Characterization: Aberration-corrected STEM characterization was performed on a ThermoFisher Themis Z microscope equipped with two aberration correctors under 300 kV. High angle annular dark field (HAADF)-STEM images were recorded using a convergence semi angle of 11 mrad, and inner- and outer collection angles of 59 and 200 mrad, respectively.

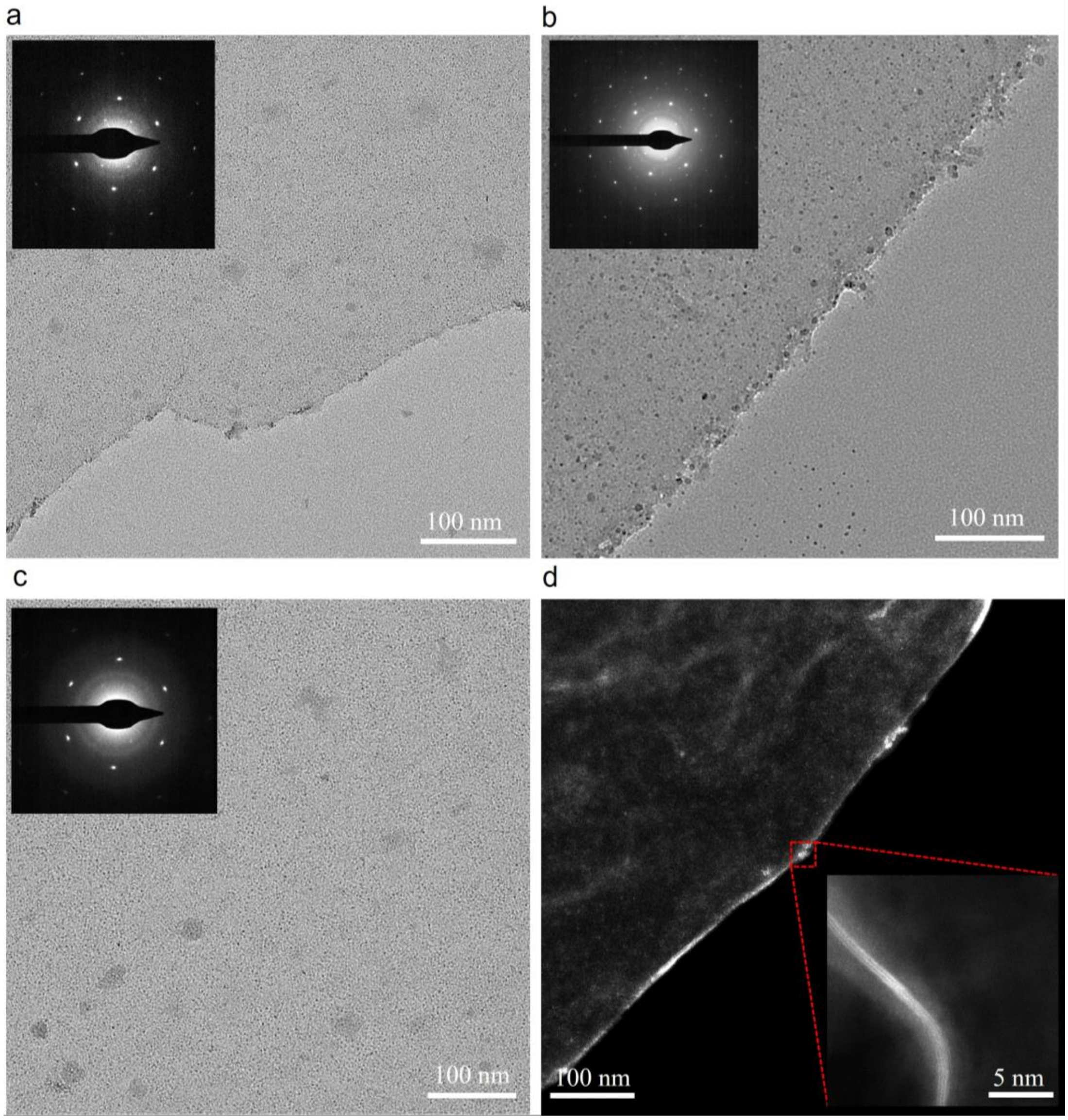

Supplementary Fig. 6 TEM and STEM images of monolayer $Ti_3C_2T_x$ MXene on a PTP device. (a, b, c) The crystal structure of tested sample on the PTP device: Low-magnification TEM image of the fracture edges, the FIB-cut edges, the center area of $Ti_3C_2T_x$ MXene, and corresponding SAED pattern, respectively. (d) Low-magnification STEM image of the fracture edges area of $Ti_3C_2T_x$ MXene. Zoom-in view of the red rectangle area shows the fractured cross-sectional surface of the tested sample.

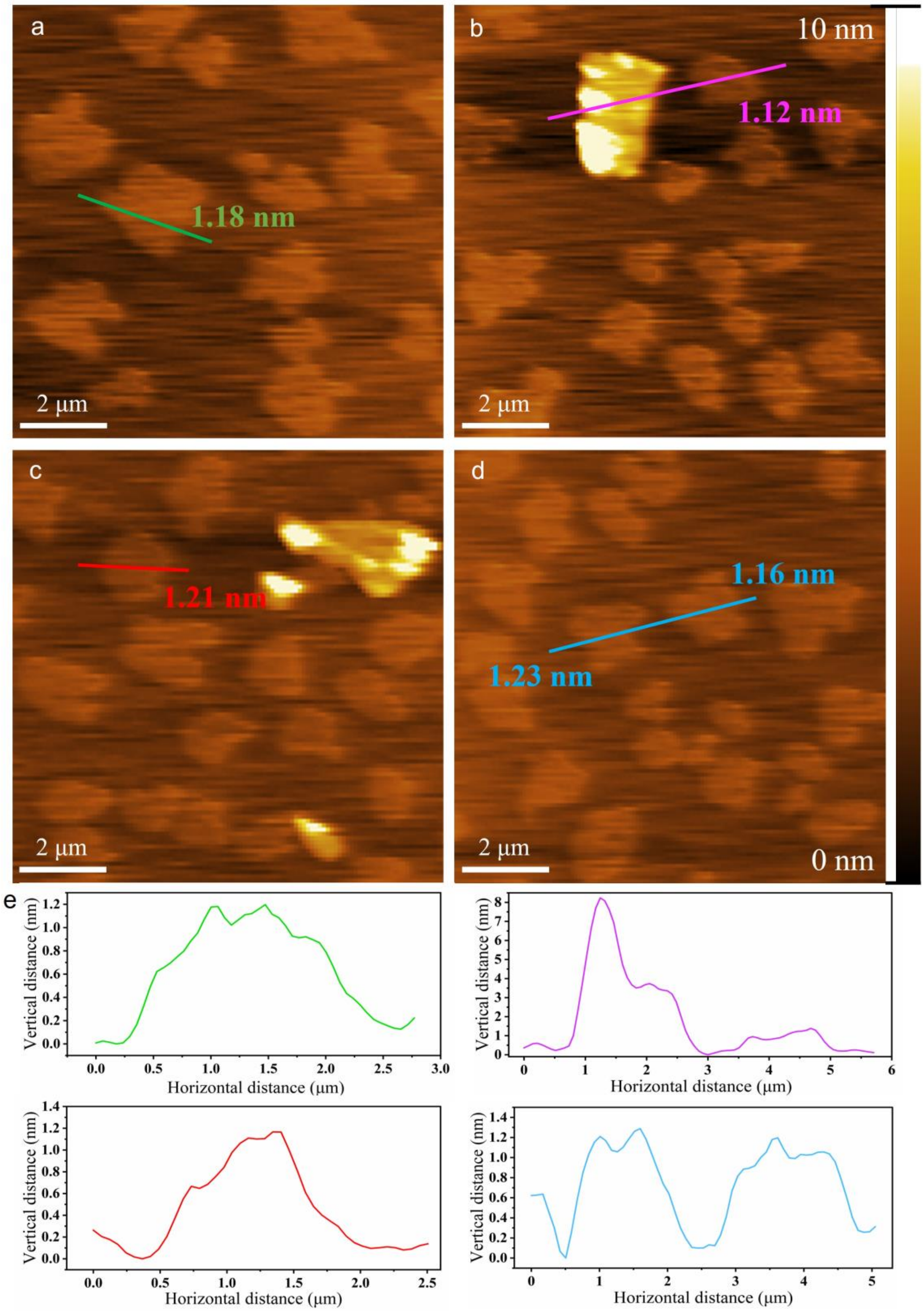

Supplementary Fig. 7 Thickness of $Ti_3C_2T_x$ MXene nanosheets. (a-d) AFM image of a large number of $Ti_3C_2T_x$ MXene nanosheets on the silicon substrate. The proportion of monolayer nanosheet is more than 95%. (e) Height profiles of MXene nanosheets in (a-d).

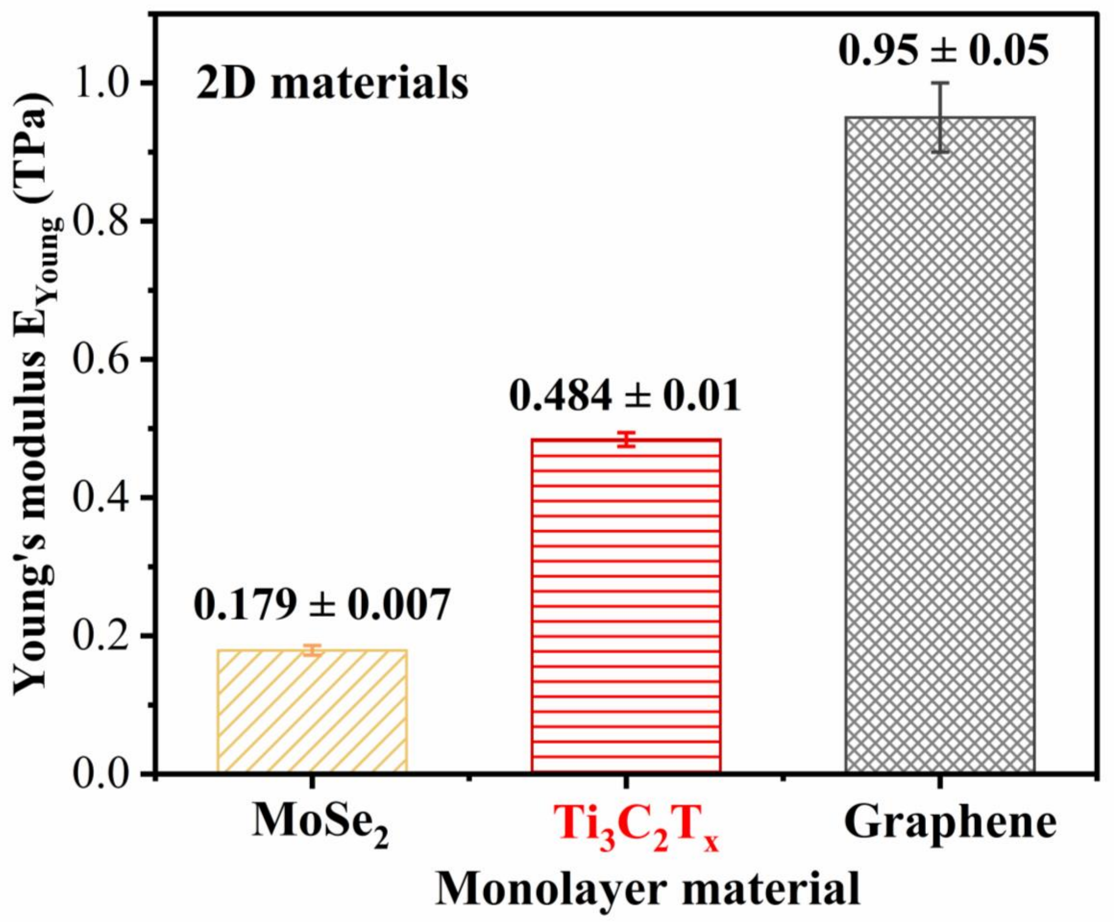

Supplementary Fig. 8 Comparison of effective Young's modulus for three 2D materials: $MoSe_2$, $Ti_3C_2T_x$, and graphene. In this chart, we compare values produced on nanosheets of monolayer 2D materials in similar PTP in-situ tensile experiments.

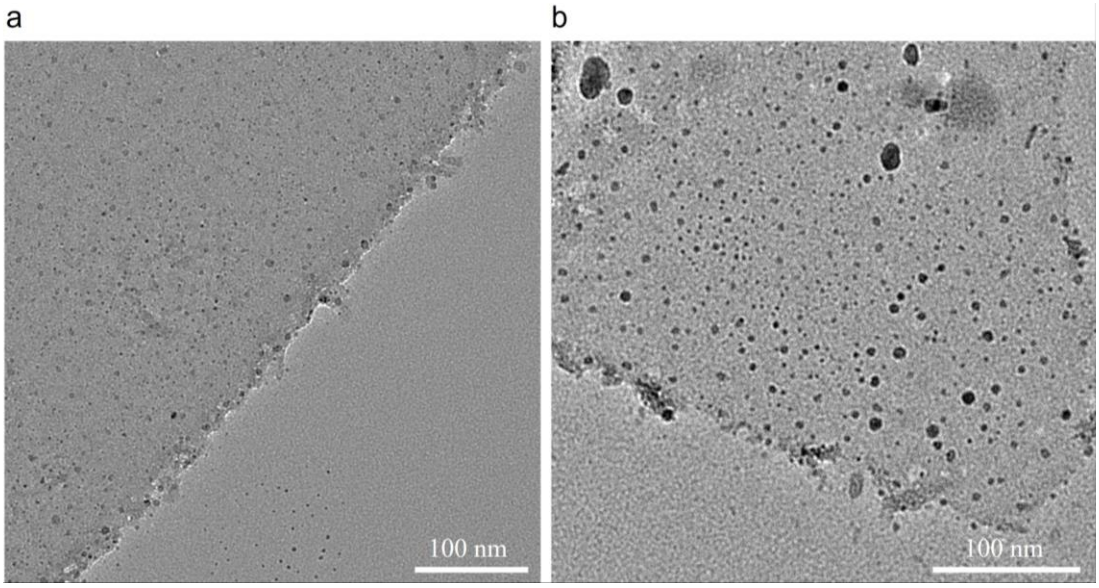

Supplementary Fig. 9 Edge defect concentration of $Ti_3C_2T_x$ MXene nanosheets by setting different FIB currents and voltages. (a) TEM images of the edges area of tested sample cut by FIB setting 2 kV, 1 pA. (b) TEM images of the edges area of the sample cut by FIB setting 2 kV, 20 pA.

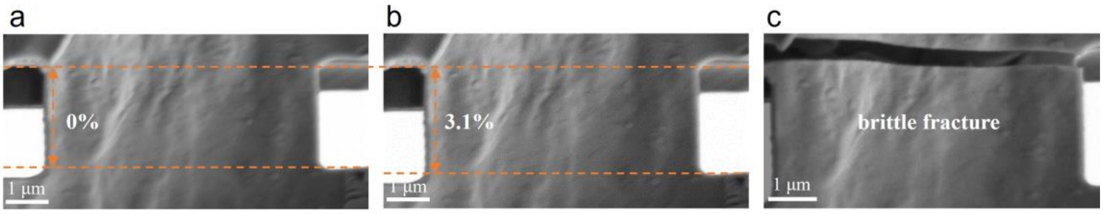

Supplementary Fig. 10 Tensile fracture of monolayer $Ti_3C_2T_x$ nanosheets redeposited with large amounts of Pt. (a) SEM image shows that the $Ti_3C_2T_x$ specimen was completely tightened at 0% strain. (b) SEM image of the sample before tensile fracture shows a peak strain of 3.1%. (c) The brittle fracture morphology of the sample after failure. The associated results are listed in Table 1 (sample #2).

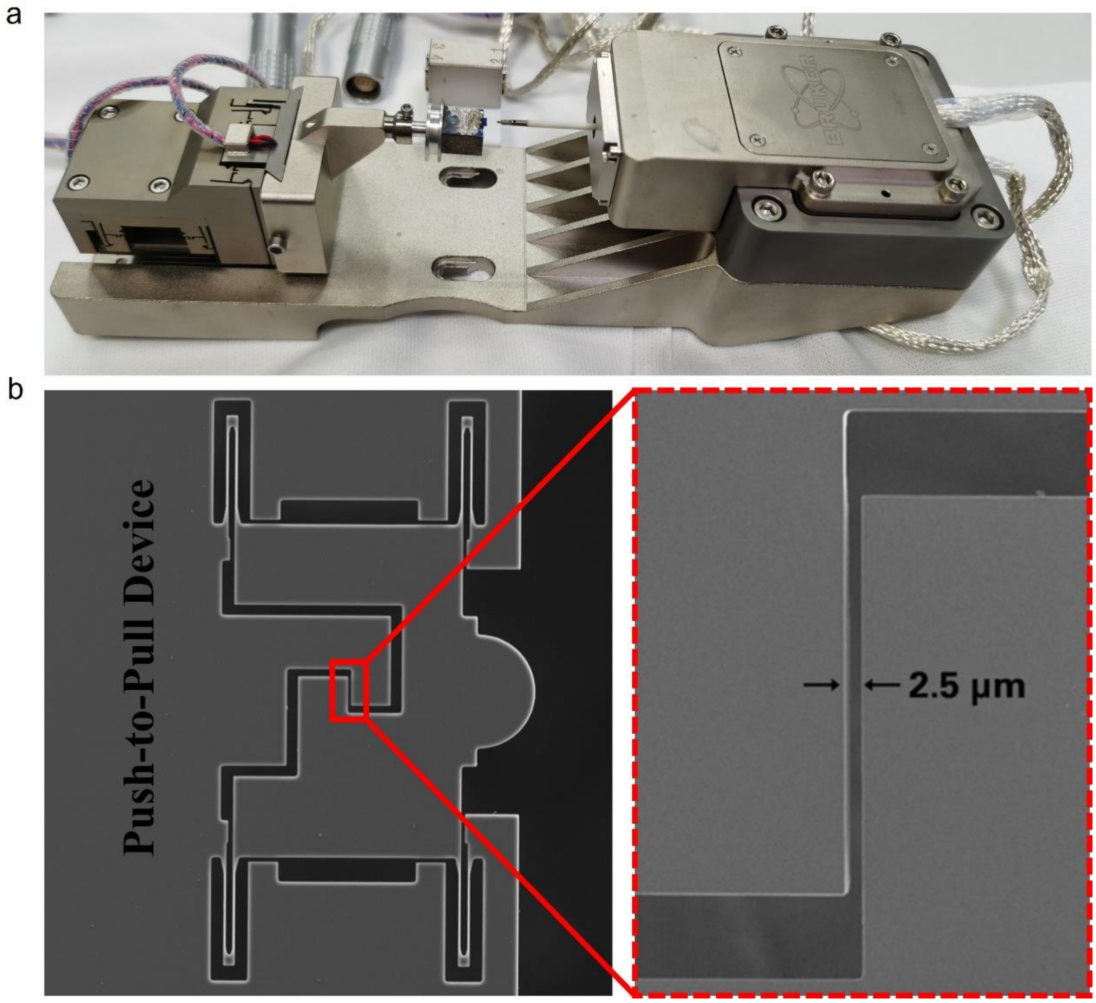

Supplementary Fig. 11 Push-to-Pull nanomechanical testing device. (a) Bruker's Hysitron PI88. Extended range in-situ nanomechanical testing instrument for scanning electron microscopes. (b) Push-to-Pull Device. Optical image of a PTP device (left) and SEM image of the PTP device gap (right).

The theoretical stiffness of the empty PTP nanomechanical devices without loading the sample is in the range of 20~100 N/m. The actual stiffness needs to be obtained from the slope of the force-displacement line at the initial stage during the test, or the slope of the tested sample after fracture. The probe is made of diamond and can apply a pushing force to the hemispherical indenter. The PTP device has four identical springs, symmetrically distributed at the corners so that the sample placed in the middle gap of the PTP device can be tensioned.

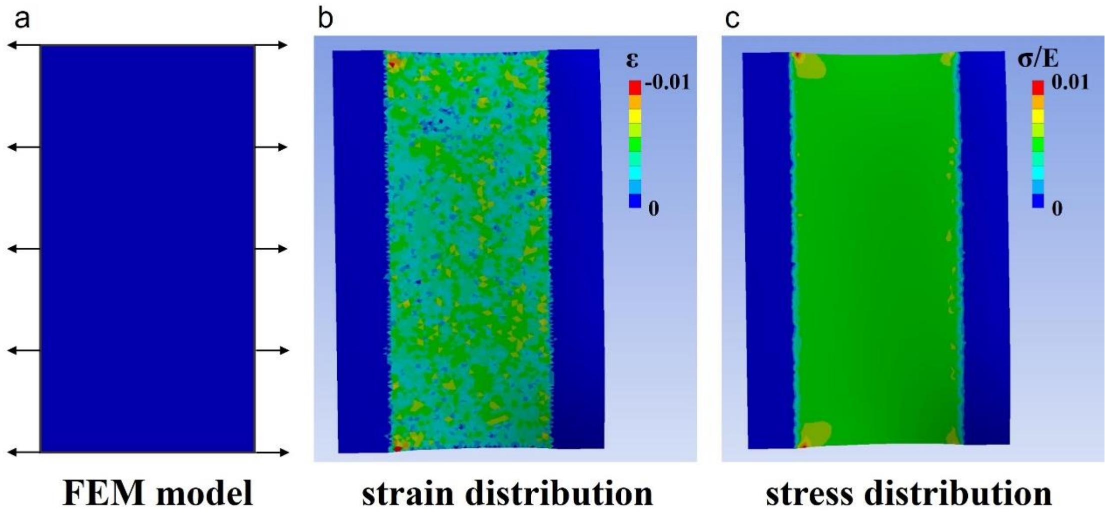

Supplementary Fig. 12 The normalized stress and strain distributions of monolayer $Ti_3C_2T_x$ MXene were predicted by finite element method (FEM). (a) The simulation setup for the monolayer $Ti_3C_2T_x$ MXene nanosheet model following the sample geometry shown in Fig. 2. The clamping end uses displacement-based loading conditions to stretch the sample. Material parameters E_{2D}=474 N/m, v=0.316 were selected according to the theoretical prediction in Reference 23. (b) Distribution of normalized strain ε, and (c) Distribution of normalized stress σ/E in the monolayer $Ti_3C_2T_x$ MXene nanosheet.

Supplementary Table 1. The thickness of monolayer $Ti_3C_2T_x$ MXene nanosheets in Supplementary Fig. 7 was statistically analyzed, and the average thickness was 1.17nm.

Sample #	Thicknesses (nm)	Sample #	Thicknesses (nm)	Sample #	Thicknesses (nm)
1	1.18	18	1.23	36	1.21
2	1.15	19	1.21	37	1.20
3	1.21	20	1.23	38	1.18
4	1.16	21	1.15	39	1.14
5	1.19	22	1.13	40	1.17
6	1.21	23	1.19	41	1.14
7	1.26	24	1.15	42	1.13
8	1.16	25	1.20	43	1.12
9	1.23	26	1.21	44	1.23
10	1.15	27	1.19	45	1.25
11	1.18	28	1.19	46	1.28
12	1.23	29	1.16	47	1.16
13	1.26	30	1.18	48	1.19
14	1.18	31	1.23	49	1.19
15	1.22	32	1.24	50	1.20
16	1.20	33	1.18	51	1.21
17	1.16	34	1.17	52	1.23
**Average thicknesses (nm)**				**1.17**	

Supplementary Table 2. Theoretical simulated fracture strength values (σ_0) for three different widths of the defect-free monolayer $Ti_3C_2T_x$ MXene nanosheets in Fig. 4.

Sample #	Length (Å)	Width (Å)	Fracture strength (GPa)
$\delta_{0\text{-}\mathrm{I}}$	122	95	18.46
$\delta_{0\text{-}\mathrm{II}}$	122	142	18.39
$\delta_{0\text{-}\mathrm{III}}$	122	190	18.41

Supplementary Table 3. The simulated theoretical fracture strength values (σ_0) for monolayer $Ti_3C_2T_x$ MXene nanosheets implanted with three edge defects of different widths in Fig. 4.

Sample #	Length (Å)	Width (Å)	Orientation	Fracture strength (GPa)
A_1	122	95	Armchair	12.42
A_2	122	95	Armchair	12.24
A_3	122	95	Armchair	11.88
Z_1	122	95	Zigzag	12.33
Z_2	122	95	Zigzag	11.61
Z_3	122	95	Zigzag	11.52
A_4	122	142	Armchair	14.45
A_5	122	142	Armchair	13.36
A_6	122	142	Armchair	12.44
Z_4	122	142	Zigzag	14.25
Z_5	122	142	Zigzag	13.21
Z_6	122	142	Zigzag	12.67
A_7	122	190	Armchair	15.58
A_8	122	190	Armchair	14.39
A_9	122	190	Armchair	13.24
Z_7	122	190	Zigzag	14.88
Z_8	122	190	Zigzag	13.52
Z_9	122	190	Zigzag	12.89

Supplementary Table 4. The measured load-displacement source data for Fig. 3d.

Number of Points = 740.

Displacement (nm) Load (μN)

0.258342	2.756994	-1.307005	0.619266	-1.938557	-1.477881	-2.744270	-2.463055
0.286910	2.480300	-0.787303	0.408136	-1.734202	-1.582625	-2.577047	-2.595359
0.081769	2.330705	-0.971143	0.407314	-2.193577	-1.492929	-3.403972	-2.347132
-0.191176	2.362601	-1.421433	0.537179	-2.283137	-1.321846	-3.235314	-2.327533
-0.035589	2.159758	-0.830577	0.288050	-1.893212	-1.495809	-2.887001	-2.367718
-0.096641	2.102129	-1.133942	0.294929	-2.222919	-1.451752	-3.085479	-2.378183
-0.654011	2.313232	-1.569943	0.484264	-2.266284	-1.457526	-3.046946	-2.445923
-0.192259	2.150144	-1.270974	0.437954	-1.999117	-1.571032	-2.943434	-2.488873
-0.124983	2.035739	-0.840952	0.213317	-1.862998	-1.695725	-3.250460	-2.398767
-0.141534	1.978986	-1.187421	0.136221	-2.101021	-1.737427	-3.124437	-2.520316
-0.658948	2.047700	-1.192203	0.032034	-2.194623	-1.807303	-2.809552	-2.712829
-0.113987	1.836958	-0.949807	-0.214119	-2.138464	-1.924675	-3.148537	-2.670864
-0.095723	1.654240	-0.863711	-0.465535	-2.533888	-1.757340	-3.333919	-2.621636
-0.585532	1.786171	-1.135158	-0.580022	-2.117837	-1.971221	-3.258786	-2.676940
-0.249923	1.536763	-1.325817	-0.628486	-2.351843	-1.885092	-3.135189	-2.720576
0.105460	1.266414	-1.252265	-0.745229	-2.509934	-1.770776	-3.551088	-2.643007
-0.767344	1.398458	-1.344248	-0.758325	-2.729101	-1.597836	-3.229696	-2.786295
-0.492662	1.262176	-1.590422	-0.808592	-2.293482	-1.771139	-3.165842	-2.810259
-0.275362	1.112010	-1.612474	-0.855737	-2.491089	-1.698800	-3.323855	-2.778164
-0.747550	1.260266	-1.309919	-0.939760	-2.567994	-1.719624	-3.623712	-2.726727
-0.813886	1.200297	-1.723461	-0.829953	-2.565530	-1.777474	-3.238954	-2.830120
-0.216296	0.994609	-1.409658	-0.955977	-2.308605	-1.833608	-3.475122	-2.821767
-0.553320	0.935474	-1.376636	-1.041967	-2.428485	-1.932318	-3.467166	-2.866408
-0.970601	1.050776	-2.017444	-0.849695	-2.856175	-1.746577	-3.379869	-2.937256
-0.956102	1.087482	-1.701441	-0.953372	-2.480613	-1.869195	-3.402209	-2.963832
-0.352299	0.858050	-1.045973	-1.387257	-2.414489	-1.951981	-3.891389	-2.785172
-0.859875	0.969534	-1.875988	-1.154486	-2.628763	-1.976146	-3.282749	-3.030529
-0.806677	0.864422	-1.943193	-1.072174	-2.699287	-1.997610	-3.381717	-3.121784
-0.544736	0.683408	-1.412721	-1.281620	-2.462406	-2.094383	-3.758178	-3.029153
-0.620264	0.610933	-1.789745	-1.265541	-2.686224	-2.051803	-3.556072	-3.157446
-1.095807	0.666977	-1.908552	-1.342544	-2.704583	-2.164263	-3.386326	-3.241399
-0.969849	0.669636	-1.602574	-1.502485	-2.432705	-2.365383	-3.896812	-3.109249
-0.690366	0.550978	-1.920414	-1.372413	-2.909302	-2.253486	-4.151156	-2.926156
-1.181570	0.707682	-1.984331	-1.356801	-2.909177	-2.229508	-3.384616	-3.252333
-0.999449	0.604224	-1.804942	-1.425056	-2.367687	-2.590994	-3.707384	-3.182597
-0.740714	0.472571	-1.919907	-1.425610	-3.014897	-2.395561	-4.040169	-3.170153
-1.218725	0.597105	-2.232395	-1.337996	-3.212168	-2.291533	-3.839007	-3.176109

-3.447457	-3.272149	-5.378157	-3.008206	-5.907930	-5.066439	-7.140831	-5.446172
-3.834044	-3.310503	-4.835750	-3.249089	-5.845824	-5.146166	-6.858632	-5.583615
-4.014289	-3.270137	-4.633862	-3.438005	-6.274650	-4.996251	-6.745194	-5.674466
-3.789642	-3.422726	-4.901976	-3.406742	-5.979699	-4.959407	-6.796275	-5.807251
-4.380421	-3.184446	-5.059006	-3.320133	-6.009330	-4.968884	-7.265944	-5.617432
-3.904310	-3.325853	-4.832186	-3.396029	-6.291500	-4.800127	-7.316139	-5.567737
-3.733269	-3.444089	-4.692818	-3.553712	-6.247525	-4.819474	-6.658515	-5.799464
-3.988130	-3.350512	-5.261403	-3.449906	-5.578931	-5.102890	-7.081088	-5.772592
-4.490222	-3.115875	-5.034380	-3.565090	-6.137010	-4.972309	-7.432375	-5.661788
-3.982464	-3.265599	-5.060402	-3.644035	-6.370387	-4.853889	-7.127834	-5.668246
-4.248776	-3.177944	-5.150416	-3.570266	-5.935033	-5.023944	-7.442672	-5.468491
-4.934746	-2.767897	-5.072617	-3.667310	-6.061951	-5.063753	-7.283222	-5.510230
-4.237813	-2.830585	-5.127789	-3.628153	-6.610498	-4.889421	-6.811714	-5.802290
-4.074158	-2.818373	-5.367324	-3.565316	-5.906961	-5.161796	-7.251202	-5.739878
-4.525576	-2.616412	-5.125378	-3.691247	-6.116431	-5.146794	-7.448598	-5.671554
-4.506902	-2.580131	-5.271836	-3.767540	-6.315407	-5.140120	-7.208646	-5.794109
-4.384405	-2.496874	-5.166396	-3.755028	-6.538206	-4.981985	-7.347396	-5.735128
-4.344115	-2.473780	-5.394482	-3.722341	-6.011330	-5.239732	-7.844037	-5.496635
-4.259266	-2.558608	-4.939087	-3.931329	-6.265936	-5.206370	-7.534279	-5.550817
-4.320066	-2.552486	-5.367411	-3.878950	-6.580584	-5.102290	-7.031431	-5.697544
-4.327156	-2.433407	-5.459816	-3.891419	-6.540226	-5.101957	-7.489449	-5.591573
-4.583205	-2.398436	-5.274072	-3.978977	-6.130413	-5.230880	-7.627956	-5.555042
-4.682623	-2.340418	-5.378714	-4.025092	-6.726127	-5.081088	-7.083523	-5.766741
-4.171141	-2.566409	-5.515763	-3.991863	-6.329719	-5.246319	-7.370811	-5.780411
-4.615861	-2.425463	-5.554117	-3.961962	-6.229046	-5.408090	-7.798787	-5.697786
-4.556934	-2.480813	-5.422138	-3.959629	-6.828143	-5.194475	-7.526234	-5.734227
-4.217784	-2.573534	-5.235113	-4.152573	-6.699358	-5.158750	-7.467379	-5.759005
-4.261535	-2.619235	-5.635614	-4.121829	-6.253098	-5.430844	-7.744572	-5.724970
-5.063634	-2.325055	-5.335486	-4.302238	-6.857123	-5.197583	-7.165560	-6.062839
-4.788327	-2.414053	-5.382776	-4.403265	-6.772877	-5.208919	-7.824346	-5.828877
-4.241702	-2.631502	-5.232944	-4.638104	-6.563654	-5.209783	-7.868905	-5.790798
-4.702181	-2.566956	-5.270297	-4.846561	-6.866513	-5.162866	-7.509313	-5.971327
-4.432893	-2.721170	-5.738165	-4.745515	-6.483800	-5.314717	-7.644338	-5.959656
-4.521464	-2.678040	-5.790782	-4.720184	-6.617199	-5.326948	-8.157779	-5.743278
-4.778342	-2.650905	-5.539130	-4.859831	-6.876304	-5.268028	-8.083862	-5.650206
-4.480204	-2.879298	-5.450838	-5.073911	-6.544022	-5.416963	-7.633266	-5.789892
-4.598965	-3.012726	-5.740124	-5.032810	-6.784714	-5.416347	-7.553704	-5.934845
-4.540507	-3.098029	-5.797766	-5.084873	-6.833744	-5.443256	-7.927715	-5.864750
-4.801341	-3.133403	-5.738758	-5.091964	-6.847634	-5.454039	-7.838521	-5.920004
-4.385606	-3.352322	-5.786632	-5.078195	-6.691756	-5.532447	-7.626876	-6.027298
-4.752000	-3.266673	-6.126012	-4.995321	-6.899173	-5.542709	-7.739413	-6.135000

-8.382625	-5.940233	-8.855474	-6.090366	-10.309156	-6.335589	-9.103607	-6.184479
-7.988591	-5.997676	-9.378531	-5.820231	-9.618387	-6.518599	-8.547621	-6.417515
-7.931340	-5.935774	-8.956209	-6.028702	-10.523185	-6.248720	-8.789729	-6.303191
-8.036569	-6.011944	-8.658378	-6.197761	-9.146709	-6.821239	-8.706776	-6.310368
-8.025339	-6.063457	-8.841360	-6.266849	-11.104155	-6.208309	-8.669229	-6.345962
-8.076590	-6.008400	-9.738807	-5.927010	-9.632734	-6.682970	-8.604079	-6.364684
-8.274114	-5.902686	-8.921661	-6.168612	-10.748254	-5.958211	-8.608934	-6.307571
-8.130585	-6.030106	-8.843414	-6.225701	-9.422390	-6.553449	-8.786173	-6.159386
-7.738075	-6.177849	-9.457396	-6.071588	-10.322650	-6.278785	-8.329421	-6.287736
-8.491409	-5.966781	-9.217092	-6.258861	-9.864775	-6.580648	-8.484596	-6.232953
-8.271077	-6.036212	-9.159294	-6.082006	-9.879091	-6.410018	-8.685962	-6.198075
-7.665908	-6.323404	-9.606208	-5.942358	-10.627034	-5.983837	-8.197968	-6.178700
-8.427993	-6.156818	-9.138089	-6.169887	-9.312663	-6.479765	-8.435102	-6.161627
-8.613539	-6.044028	-9.184285	-6.135537	-10.096516	-6.214163	-8.406960	-6.119696
-8.301375	-6.115532	-9.503954	-6.102184	-10.111004	-6.175305	-8.549879	-5.886137
-7.999083	-6.327319	-9.239881	-6.209018	-9.053688	-6.483767	-8.510382	-5.577548
-8.590677	-6.062388	-9.440544	-6.229064	-10.571290	-5.852882	-8.101403	-5.678562
-8.698403	-5.993847	-9.468072	-6.058842	-9.145268	-6.476227	-8.300271	-5.540840
-8.717482	-5.851335	-10.256352	-5.647866	-9.492705	-6.481615	-8.264310	-5.463009
-8.493919	-5.849828	-9.019438	-6.072683	-9.838760	-6.299105	-8.241535	-5.363191
-8.334759	-5.928965	-9.083266	-6.198612	-9.620883	-6.383526	-8.149849	-5.205871
-8.334183	-6.054409	-9.528473	-6.114608	-9.665896	-6.281750	-8.024388	-5.166065
-8.653181	-5.921273	-9.884479	-6.009592	-10.335716	-5.895543	-7.979606	-5.145386
-8.357688	-6.052356	-9.419458	-6.164515	-8.531179	-6.422222	-8.036184	-5.175678
-8.784194	-5.923720	-8.842339	-6.504035	-9.959513	-6.142946	-7.736016	-5.211829
-8.467639	-6.043784	-10.977360	-5.721441	-8.798207	-6.473825	-8.158441	-4.999268
-8.752611	-5.911209	-9.341418	-6.118082	-9.817221	-5.984078	-7.512773	-5.321456
-8.728869	-5.861003	-8.961996	-6.297246	-9.087295	-6.272469	-7.809524	-5.280404
-8.531877	-5.968748	-10.295939	-5.943076	-9.250838	-6.368458	-7.642389	-5.353585
-8.992287	-5.774519	-9.794410	-6.117932	-9.584921	-6.081761	-7.794784	-5.346787
-9.044125	-5.676972	-9.313465	-6.151268	-8.976016	-6.329570	-7.793647	-5.278263
-8.413246	-5.895089	-10.189189	-5.931849	-9.757599	-5.976964	-7.452431	-5.438470
-8.805688	-5.822573	-9.657532	-6.213175	-8.774146	-6.211799	-7.654987	-5.382197
-8.758627	-5.869471	-9.204158	-6.466375	-9.163573	-6.109197	-7.572545	-5.443503
-8.962283	-5.771598	-10.293549	-6.137396	-9.165367	-6.127083	-7.610289	-5.289637
-8.911092	-5.769982	-9.806589	-6.286589	-8.808341	-6.163679	-7.687695	-5.209822
-8.607512	-5.942583	-8.927698	-6.799242	-9.140072	-6.127465	-7.436473	-5.170157
-9.125822	-5.866326	-10.755424	-6.151102	-8.798638	-6.234265	-7.413067	-5.154530
-8.399043	-6.110254	-10.236166	-6.228234	-8.764800	-6.363158	-7.331016	-5.189966
-9.042838	-5.873460	-9.304335	-6.512097	-9.415774	-6.063547	-7.264981	-5.287573
-8.951702	-6.005625	-10.119496	-6.368835	-8.308583	-6.401107	-7.578300	-5.066590

-7.065070	-5.272825	-5.994573	-4.300004	-4.390417	-1.706647	-2.852939	-0.927287
-7.393952	-5.030997	-5.719902	-4.377116	-4.167534	-1.746794	-2.664633	-0.955892
-7.238386	-5.148468	-5.571670	-4.342226	-4.060186	-1.804299	-2.832725	-0.773689
-7.133939	-5.070505	-5.865913	-4.170458	-4.112979	-1.772367	-2.621613	-0.607841
-6.821108	-5.399576	-5.575479	-4.227246	-4.050941	-1.840749	-2.350621	-0.825583
-7.089926	-5.498258	-5.455429	-4.189594	-3.953024	-1.861326	-2.547188	-0.706527
-6.863481	-5.587237	-5.665371	-4.061937	-4.176650	-1.730879	-2.424122	-0.818433
-7.344814	-5.327887	-5.567526	-4.015491	-3.551647	-1.970312	-2.301505	-0.795477
-6.812738	-5.482533	-5.396740	-4.017285	-3.836002	-2.218649	-2.312273	-0.948400
-7.136674	-5.232750	-5.516398	-4.015832	-3.500164	-2.450549	-2.322765	-0.909568
-6.971212	-5.218276	-5.379707	-3.970406	-3.843684	-2.446165	-2.339762	-0.870410
-6.665120	-5.285807	-5.339318	-3.959794	-3.867900	-2.436081	-2.453384	-0.608371
-6.717867	-5.404829	-5.346586	-3.819326	-3.663734	-2.500025	-2.227288	-0.654697
-6.872081	-5.387233	-5.250360	-3.888239	-3.679451	-2.529650	-2.356213	-0.534405
-6.830997	-5.301244	-5.357257	-3.776952	-3.902193	-2.368809	-2.090921	-0.515332
-6.791999	-5.310010	-5.010771	-3.917866	-3.640599	-2.269810	-2.415228	-0.191178
-6.590256	-5.296717	-5.431457	-3.597231	-3.706656	-2.169925	-2.048209	-0.253369
-6.816292	-5.076141	-5.233628	-3.484868	-3.441139	-2.302001	-2.261055	0.002894
-6.612490	-5.110083	-5.590496	-3.009494	-3.598007	-2.244398	-2.003453	-0.000542
-6.779578	-4.941179	-5.082640	-2.975087	-3.572285	-2.176549	-2.013761	0.023136
-6.497241	-4.978259	-5.116360	-2.751809	-3.617443	-2.020725	-1.881293	0.059719
-6.449979	-4.976218	-5.171554	-2.586982	-3.517153	-1.937813	-2.384071	0.366072
-6.532617	-4.818648	-4.896235	-2.568570	-3.377176	-1.900148	-2.004894	0.705519
-6.394678	-4.881763	-4.949258	-2.501136	-3.570136	-1.689942	-1.914184	0.778158
-6.573902	-4.622365	-5.170194	-2.320573	-3.285793	-1.658831	-1.972077	1.017501
-6.278371	-4.718816	-4.567299	-2.359305	-3.337492	-1.629262	-1.903964	1.110298
-6.202043	-4.805641	-5.225686	-2.006836	-3.308067	-1.554626	-1.691604	1.291216
-6.426014	-4.705990	-4.653198	-2.047807	-3.190908	-1.506319	-1.767625	1.362200
-6.145156	-4.730014	-4.709906	-1.982246	-3.118646	-1.519358	-1.778726	1.468001
-6.459649	-4.460511	-4.615139	-2.055115	-3.257077	-1.433021	-1.888704	1.744030
-6.220902	-4.419912	-4.496774	-2.146545	-2.874289	-1.599361	-1.652206	1.869636
-5.928802	-4.527245	-4.678838	-2.064758	-3.230751	-1.442592	-1.439846	1.745076
-6.062364	-4.561645	-4.555901	-2.109895	-2.947878	-1.414083	-1.492532	1.836788
-6.062509	-4.591467	-4.165570	-2.323043	-3.072702	-1.386501	-1.423793	1.845912
-6.066670	-4.509826	-4.696832	-2.166286	-2.967852	-1.386772	-1.324395	1.841320
-5.856507	-4.591980	-4.417038	-2.140480	-2.900570	-1.279872	-1.359926	1.747060
-6.233972	-4.398634	-4.400768	-2.171384	-2.738533	-1.393400	-1.264455	1.704709
-5.739854	-4.434847	-4.376462	-2.047251	-2.972961	-1.335342	-1.127897	1.591724
-5.875876	-4.387680	-4.455275	-2.036013	-2.820254	-1.176803	-1.363716	1.608515
-5.774774	-4.490961	-4.392233	-1.841339	-2.903784	-1.128682	-1.038735	1.536488
-5.682201	-4.550952	-4.153200	-1.930273	-2.619263	-1.090169	-1.406250	1.789702

-1.274841	1.868162	62.202423	1.672844	1957.151899	55.611085
-0.972038	1.794367	71.504982	2.269464	1968.432423	55.849204
-1.190500	1.906113	82.183191	2.340470	1977.734982	56.445824
-0.998214	1.857844	95.237067	2.584349	1988.413191	56.516830
-0.987339	1.881029	106.828717	3.157980	2001.467067	56.760709
-1.210073	2.055396	118.416959	3.507900	2013.058717	57.334340
-0.790241	2.122291	130.356513	3.768585	2024.646959	57.684260
-1.043476	2.153117	145.018690	4.266365	2036.586513	57.944945
-0.654074	2.031480	159.516669	4.617605	2051.248690	58.442725
-0.949069	2.134931	172.693049	5.151667	2065.746669	58.793965
-0.403621	1.841081	188.238685	5.536014	2078.923049	59.328027
-0.414381	1.548767	205.309906	5.943809	2094.468685	59.712374
-0.568473	1.464320	222.014785	6.616860	2111.539906	60.120169
-0.646763	1.382726	236.995736	7.186114	2128.244785	60.793220
-0.579728	1.454141	253.943387	7.811352	2143.225736	61.362474
-0.713159	1.461937	272.057675	8.259836	2160.173387	61.987712
-0.390119	1.364776	294.412910	8.894958	2178.287675	62.436196
-0.688646	1.535106	317.730993	9.878517	2200.642910	63.071318
-0.425463	1.564005	342.265550	10.536782		
-0.406171	1.535679	369.721048	11.602797		
-0.372340	1.538589	401.903686	12.651789		
-0.381184	1.580141	437.021703	14.091724		
-0.325372	1.579750	476.921765	15.590892		
-0.231901	1.555745	527.471628	17.454560		
-0.465047	1.777721	591.917876	20.283411		
-0.177840	1.786931	676.984335	24.238201		
-0.161112	1.780311	799.623258	30.774994		
-0.191239	1.783663	998.967358	43.707096		
-0.203916	1.972681	1393.091642	78.192469		
0.087934	1.888628	1509.234739	190.129235		
-0.204261	1.985251	1665.351013	338.766469		
-0.030934	2.073671	1869.451902	534.369342		
-0.089482	2.181800	1922.243325	583.232566		
0.077621	2.199734	1922.285744	583.991536		
-0.071546	2.358555	1922.578010	584.740885		
0.000628	0.006920	1906.049000	50.176360		
10.534641	0.195039	1906.230628	51.183280		
21.055996	0.502691	1916.764641	52.371399		
29.021440	0.994559	1927.285996	53.679051		
40.681581	0.975975	1935.251440	54.170919		
50.921899	1.434725	1946.911581	55.152335		

Supplementary References

1. Halim, J., Cook, K. M., Naguib, M., Eklund, P., Gogotsi, Y., Rosen, J., Barsoum, M. W. X-Ray Photoelectron Spectroscopy of Select Multi-Layered Transition Metal Carbides (MXenes). *Appl. Surf. Sci*. **362,** 406–417 (2016).

2. Pazniak, A., Bazhin, P., Shplis, N., Kolesnikov, E., Shchetinin, I., Komissarov, A., Polcak, J., Stolin, A., Kuznetsov, D. $Ti_3C_2T_x$ MXene Characterization Produced from SHS-Ground Ti_3AlC_2. *Mater. Des*. **183,** 108143 (2019).
